# Three novel circRNAs upregulated in tissue and plasma from hepatocellular carcinoma patients and their regulatory network

**DOI:** 10.1186/s12935-021-01762-w

**Published:** 2021-01-22

**Authors:** Lianghai Wang, Lisha Zhou, Jun Hou, Jin Meng, Ke Lin, Xiangwei Wu, Xueling Chen

**Affiliations:** Key Laboratory of Xinjiang Endemic and Ethnic Diseases/the First Affiliated Hospital, Shihezi University School of Medicine, Shihezi, Xinjiang China

**Keywords:** Liver cancer, ceRNA, Biomarker, Prognosis

## Abstract

**Background:**

The regulatory roles of circular RNAs (circRNAs) in tumorigenesis have attracted increasing attention. However, novel circRNAs with the potential to be used as serum/plasma biomarkers and their regulatory mechanism in the pathogenesis of hepatocellular carcinoma (HCC) remain explored.

**Methods:**

CircRNA expression profiles of tumor tissues and plasma samples from HCC patients were compiled and jointly analyzed. CircRNA–miRNA–mRNA interactions were predicted by bioinformatics tools. The expression of interacting miRNAs and mRNA was verified in independent datasets. Survival analysis and pathway enrichment analysis were conducted on hub genes.

**Results:**

We identified three significantly up-regulated circRNAs (hsa_circ_0009910, hsa_circ_0049783, and hsa_circ_0089172) both in HCC tissues and plasma samples. Two of them were validated to be indeed circular and could be excreted from hepatoma cells. We further revealed four miRNAs (hsa-miR-455-5p, hsa-miR-615-3p, hsa-miR-18a-3p, hsa-miR-4524a-3p) that targeting circRNAs and expressed in human HCC samples, and 95 mRNAs targeted by miRNAs and significantly up-regulated in two HCC cohorts. A protein-protein interaction network revealed 19 hub genes, 12 of them (MCM6, CCNB1, CDC20, NDC80, ZWINT, ASPM, CENPU, MCM3, MCM5, ECT2, CDC7, and DLGAP5) were associated with reduced survival in two HCC cohorts. KEGG, Reactome, and Wikipathway enrichment analysis indicated that the hub genes mainly functioned in DNA replication and cell cycle.

**Conclusions:**

Our study uncovers three novel deregulated circRNAs in tumor and plasma from HCC patients and provides an insight into the pathogenesis from the circRNA–miRNA–mRNA regulatory network.

## Background

Hepatocellular carcinoma (HCC) accounts for approximately 90 % of primary liver cancer, the sixth most commonly diagnosed tumor and the fourth leading cause of cancer-related death worldwide [1]. Due to the early asymptomatic, most HCC patients are diagnosed at advanced stages with metastasis, thus losing the opportunity for radical resection [2, 3]. With the improvement of diagnosis and treatment technologies, the survival of HCC patients has increased. However, the 5-year survival rate is only 18 % as high frequencies of tumor metastasis and recurrence [1]. To improve patients’ survival and quality of life, it is urgent to explore novel biomarkers for early diagnosis, prognosis, and treatment of liver cancer [2, 4, 5].

Circular RNA (circRNA) is a type of stable transcripts that do not have a free 5′ end cap and 3′ end poly (A) tail and forms a circular structure by covalent bonds [6, 7]. CircRNA was initially considered to be a by-product of splicing. Nevertheless, with the advancement of high-throughput sequencing technology and bioinformatics analysis, the cellular functions and potential biomedical applications of circRNAs become a new research hotspot [8]. CircRNAs are abundant in the cytoplasm of eukaryotic cells and cell-type specific [9–11]. Recent studies have shown that circRNAs are essential to modulate gene expression in the nucleus, act as decoys for miRNAs and proteins, and serve as templates for translation [3, 12–15]. The regulatory roles of circRNAs in tumorigenesis have also attracted more attention [5, 16, 17]. Increasing studies suggest that circRNAs play essential roles in the occurrence and development of HCC, and are expected to have the potential to become diagnostic biomarkers and therapeutic targest [18, 19]. However, novel circRNAs with the potential to be used as serum/plasma biomarkers and their regulatory mechanism in liver cancer pathogenesis remain to be explored.

**Fig. 1:**
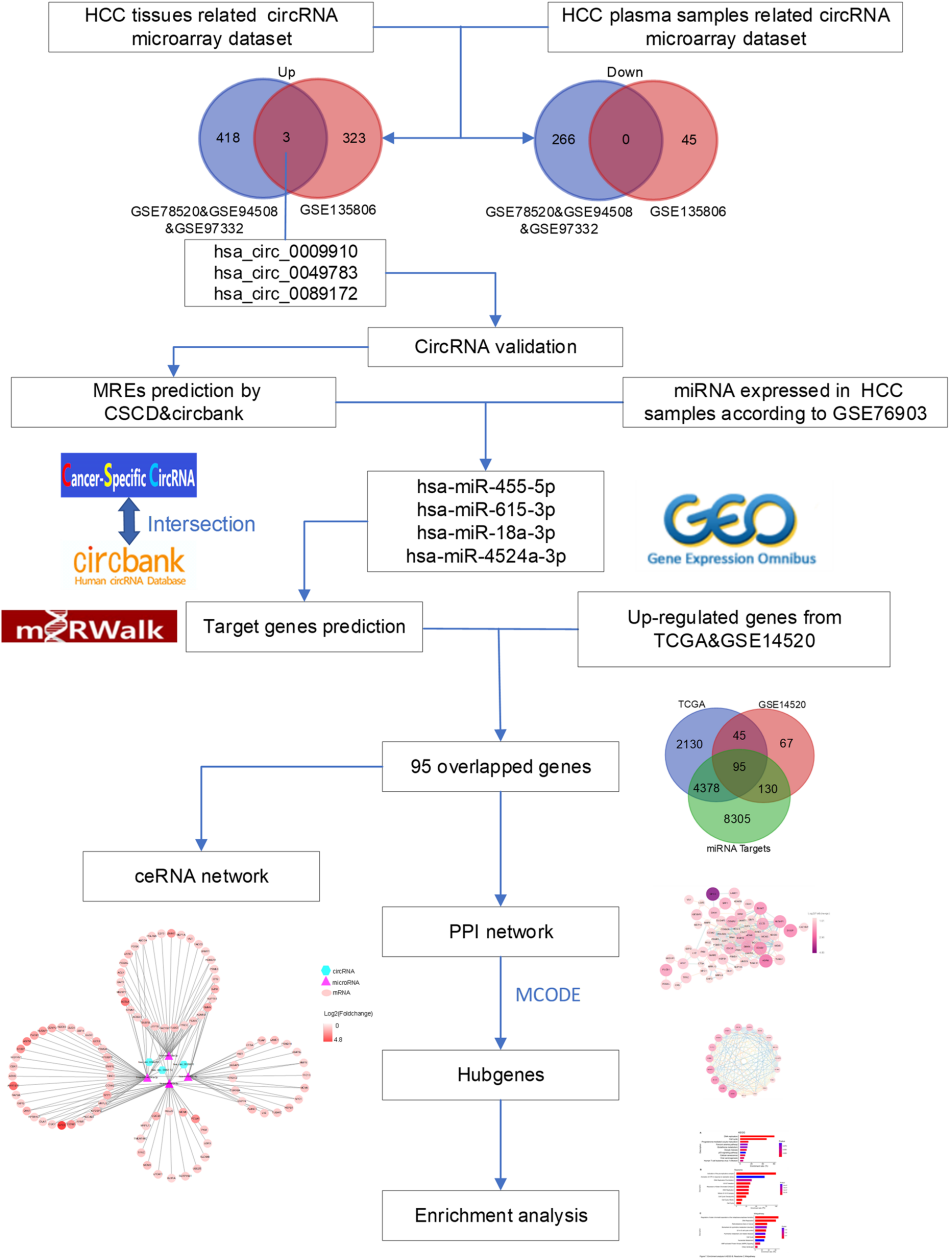
Flow chart of the present study

In this study, we jointly analyzed three HCC tissues-related microarray datasets from the Gene Expression Omnibus (GEO). Such a relatively large sample size allows for higher statistical power in the identification of differentially expressed circRNAs. CircRNAs that were also increased in HCC plasma were obtained by overlapping with an HCC plasma samples-related microarray dataset. Subsequently, a circRNA-miRNA-mRNA network was constructed to provide an insight into the potential pathogenesis of the identified circRNAs in HCC. The flow chart recapitulating the present work is shown in Fig. 1.

## Methods

### CircRNA expression processing of HCC microarray datasets

The four publicly available HCC-related circRNA microarray datasets used in this study were downloaded from the GEO database, including three gene expression profiles on platform GPL19978 and one from the platform GPL26925. Data normalization and combination were performed by pooling the three datasets of the GPL19978 platform using the Bioconductor Limma package. The principal component analysis was performed to ensure a minimal batch effect in the meta-cohort. Then, differential analysis of the pooled expression data was also conducted by Limma with the criteria of |log2(foldchange)| ≥ 0.585 and *P*-value < 0.05. Subsequently, the processed plasma circRNA data of GSE135806 based on the thresholds of |log2(foldchange)| ≥ 1 and *P*-value < 0.05 was downloaded from the GEO, and further intersected with the differentially expressed circRNAs in HCC tissues with an online tool (http://bioinformatics.psb.ugent.be/webtools/Venn/).

### Cells and cell culture

Human HCC cell lines HepG2, Huh7, and Hep3B were purchased from the Cell Bank of Shanghai Institutes for Biological Sciences, Chinese Academy of Sciences. Cells were cultured in Dulbecco’s modified Eagle’s medium (DMEM; Gibco) supplemented with 10 % fetal bovine serum (Gibco) and 1 % penicillin/streptomycin at 37 °C in a humidified 5 % CO_2_ incubator.

### CircRNA validation

The culture medium of hepatoma cells was collected, centrifuged at 1,000*g* for 10 min and 14,000*g* for 2 min to remove cells and debris [20]. Total RNA from cells or culture medium was extracted using Total RNA Kit I (OMEGA) or TRIzol LS Reagent (Invitrogen) according to the manufacturer’s instructions. cDNA was synthesized from total RNA treated with or without RNase R exonuclease (Geneseed) for real-time PCR on a CFX96 system (Bio-Rad). PCR products were further visualized by agarose gel electrophoresis. β-actin was used as an endogenous control. Primer sequences were shown in Table 1.

**Table 1:** Primer sequences for real-time PCR

Gene	Forward primer	Reverse primer
hsa_circ_0009910 (divergent)	GTGAGAGGCATCAGTGAGGT	CGAGAGAAGAGCAGGGACAT
hsa_circ_0009910 (convergent)	GGACCCCGTTACCACAGAAG	CCACTTTCATGTGCCTCCGA
hsa_circ_0089172 (divergent)	AAGAATAACCCTGCAACCCCTT	GACAGATGACGTTGAGCTGACT
hsa_circ_0089172 (convergent)	GCCTGTCTCGATCAGCCTTT	CTGAACCACTGCCTCGTCAT
β-actin	GAGAAATCTGGCACCACACC	GGATAGCACAGCCTGGATAGCAA

### Prediction of miRNA binding and miRNA target genes

The Cancer-specific circRNAs database (CSCD) [21] and circbank (http://www.circbank.cn/index.html) were used to predict miRNA response elements (MRE or binding sites). Overlapped miRNAs in the two databases were considered as potential target miRNAs of circRNAs. The expression of the candidate miRNAs was further verified base on the miRNA-seq dataset GSE76903 [22]. The miRNA–mRNA interactions were predicted with miRWalk [23], with only the results from binding to 3′ UTR filtered.

### Collection of differently expressed genes in HCC

RNA-Seq data of the TCGA-LIHC project consisting of 374 liver cancers and 50 normal tissues were downloaded from the GDC Data Portal. The DESeq2 package was used to screen differentially expressed genes with thresholds of |log 2 (fold change)| ≥ 1 and *P*-value < 0.01. With the same filter criteria, differently expressed genes from expression profiling by an array (GSE14520, GPL3921 Affymetrix HT Human Genome U133A Array) containing 225 HBV-related HCC and 220 non-tumor tissues were also determined.

### Establishment of circRNA–miRNA–mRNA network and identification of hub genes

A circRNA–miRNA–mRNA regulatory network was established by integrating the circRNA-miRNA and miRNA-mRNA interactions and visualized using the Cytoscape 3.7.2 software [24]. The candidate mRNAs were imported to the STRING database (https://string-db.org), and protein-protein interaction (PPI) network was created. Then, MCODE, a clustering algorithm that detects densely connected regions in large protein interaction networks [25], in Cytoscape was used to identify hub genes in the PPI network. The highest-scoring nodes recognized as hub genes are listed in Table 2.

**Table 2:** Basic information of 19 significantly up-regulated hub genes in TCGA-LIHC and GSE14520-GPL3921

Hub genes	MCODE Score	Log2(Fold Change)		Log-rank *P* value of overall survival	
		TCGA-LIHC	GSE14520	TCGA-LIHC	GSE14520
NUSAP1	16.50292398	1.60	2.37	0.0081**	0.1060
CCNA2	16.50292398	1.08	1.28	0.0012**	0.0514
MCM6	16.50292398	1.01	2.02	0.0003***	0.0124*
CCNB1	16.50292398	1.00	2.49	0.0001***	0.0021**
CDC20	16.50292398	1.58	1.90	< 0.0001****	0.0459*
BUB1B	16.50292398	4.74	1.71	0.0032**	0.0666
NDC80	16.50292398	4.46	1.31	0.0025**	0.0444*
ZWINT	16.50292398	1.14	2.43	< 0.0001****	0.0056**
ASPM	16.50292398	1.02	3.01	0.0172*	0.0014**
CENPU	15.58169935	1.81	1.96	0.0020**	0.0113*
FANCI	15.58169935	2.21	1.19	0.0132*	0.1117
GMNN	15.58169935	1.57	2.13	0.3349	0.0195*
HELLS	15.58169935	2.58	1.05	0.0204*	0.0554
MCM3	15.58169935	1.95	1.48	0.0261*	0.0333*
MCM5	15.58169935	2.10	1.38	0.0039**	0.0015**
ECT2	15.58169935	4.73	2.03	0.0050**	0.0254*
CDC7	15.58169935	3.75	1.09	0.0002***	0.0357*
RRM1	15.58169935	1.24	1.12	0.0294*	0.0613
DLGAP5	15.58169935	3.32	1.31	0.0003***	0.0320*

### Survival analysis of hub genes

Survival data of patients from TCGA-LIHC and patients from GSE14520 were obtained from UCSC Xena (https://xenabrowser.net/) and GEO. Patients were divided into two groups based on the median expression values for TCGA-LIHC and based on the minimum *P*-value cut off values for GSE14520. The Kaplan-Meier’s survival curves for the hub genes were visualized using GraphPad Prism.

### Functional enrichment analysis of hub genes

KEGG, Reactome, and Wiki pathway enrichment analysis of the 12 hub genes were carried out using Webgestalt online tool (http://www.webgestalt.org/). A *P*-value of less than 0.05 was set as the cut-off criterion.

### Statistical analysis

Statistical analysis was performed using GraphPad Prism (version: 8.0.2) and R software. The survival curves were calculated using the Kaplan-Meier method, and a log-rank test assessed the differences. Statistical significance was indicated by *P* values less than 0.05. *P < 0.05, ** *P* < 0.01, *** *P* < 0.001, **** *P* < 0.0001.

## Results

### Three circRNAs were significantly up‐regulated both in HCC tissues and plasma samples

**Fig. 2:**
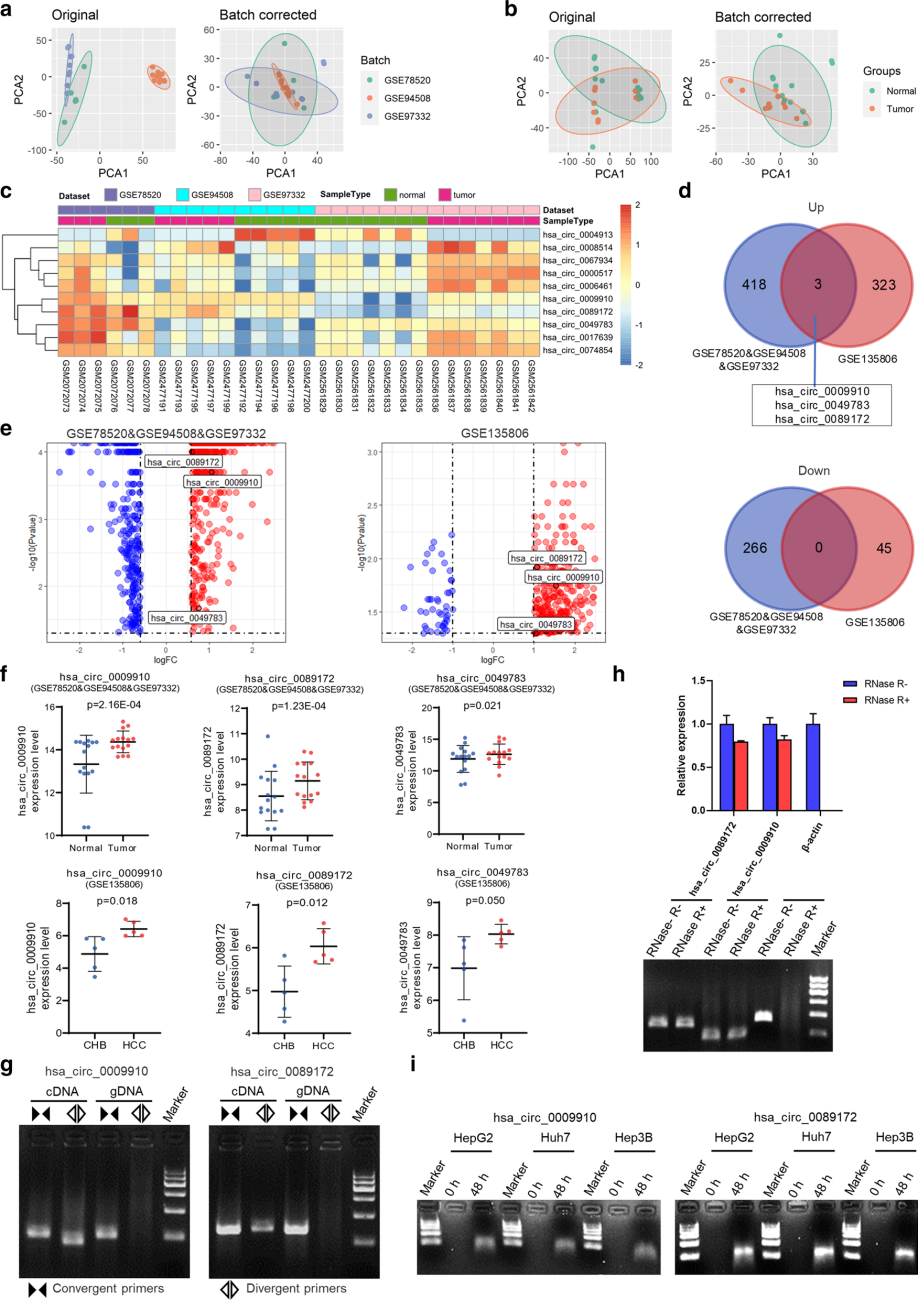
Identification of three up-regulated circRNAs in HCC tissues and plasma samples**.** **a, b **PCA plot of the first two components before (left) and after (right) Limma standardization, which minimizes the batch effect. **c** Heatmap for ten selected differentially expressed circRNAs in the pooled 15 pairs of HCC samples.** d** Venn diagram of up-regulated and down-regulated circRNAs in the HCC tissues-related meta-cohort (GSE78520&GSE94508&GSE97332) and the HCC plasma samples-related microarray dataset (GSE135806). **e** Volcano plot showing three up-regulated circRNAs in HCC based on datasets GSE78520&GSE94508&GSE97332 and GSE135806, respectively. **f** Expression of the three identified circRNAs in the respective datasets. CHB, chronic hepatitis B patients. Data were shown as mean ± SEM. **g** Agarose gel electrophoresis showed that divergent primers amplified hsa_circ_0009910 (left) and hsa_circ_0089172 (right) in cDNA but not genomic DNA (gDNA) from HepG2 cells. **h** Real-time PCR (top) and agarose gel electrophoresis (bottom) showed that hsa_circ_0009910 and hsa_circ_0089172 but not β-actin from HepG2 cells were insensitive to RNase R treatment. **i** Detection of hsa_circ_0009910 (left) and hsa_circ_0089172 (right) excretion in the indicated hepatoma cells’ culture medium

To identify differentially expressed circRNAs in HCC tissues and plasma samples, four publicly available microarray datasets providing circRNA expression profile (GSE78520, GSE94508, GSE97332, and GSE135806) were collected in this study. GSE78520, GSE94508, and GSE97332 were HCC tissues-related and were all from platform GPL19978 Agilent-069978 Arraystar Human CircRNA microarray V1, whereas GSE135806 was HCC plasma samples-related and was from the platform of GPL26925 Agilent-084217 CapitalBio Technology Human CircRNA Array v2. A summary of the four datasets was presented in Table 3. First, we performed a quality check, cohort-specific normalization, and combined the three HCC tissues-related datasets by Limma, resulting in a meta-cohort of 15 pairs of HCC and matched non-tumor tissues (GSE78520&GSE94508&GSE97332). The principal component analysis plot indicated that the batch effect due to cohort and processing centers was effectively minimized while retaining the disease state’s differences in the meta-cohort (Fig. 2a, b). A total of 687 differentially expressed circRNAs (421 up-regulated and 266 down-regulated) were identified in the pooled 15 pairs of samples (Fig. 2c, d). Previously reported differentially expressed circRNAs, including hsa_circ_0004913, hsa_circRNA_0008514, hsa_circ_0067934, hsa_circRNA_0006461, hsa_circ_0017639, and hsa_circ_0074854 [26, 27] were also identified in our analysis (Fig. 2c), proving the robustness of the above HCC meta-cohort compilation. On the other hand, a total of 371 circRNAs were differentially expressed (326 up-regulated and 45 down-regulated) in HCC plasma samples (GSE135806 [28]). Venn diagrams and volcano plots showed three circRNAs (hsa_circ_0009910, hsa_circ_0049783, and hsa_circ_0089172) were consistently up-regulated, and none were down-regulated in the integrated datasets (Fig. 2d, e). Scatter plots confirmed the increased expression of these three circRNAs in HCC tissues and plasma samples compared with their respective controls (Fig. 2f). The essential characteristics of the three identified circRNAs are displayed in Table 4. Intriguingly, regulation directions of their parent genes were different from the significantly up-regulated circRNAs, indicating that the three increased circRNAs in HCC were not by-products of splicing but were suggestive of functionality [29].

**Table 3:** Basic information of datasets used in this study

Dataset	Platform	Sample size (tumor/non-tumor)	Race	Data type	Cut-off
GSE78520	GPL19978	3/3	Chinese	CircRNA expression profile in HCC and matched non-tumor tissues	|log2(foldchange)| ≥ 0.585, *P* < 0.05
GSE94508	GPL19978	5/5	Chinese		
GSE97332	GPL19978	7/7	Chinese		
GSE135806	GPL26925	5/5	Chinese	CircRNA expression in the plasma from HBV-related HCC and chronic hepatitis B patients	|log2(foldchange)| ≥ 1, *P* < 0.05
GSE14520	GPL3921	225/220	Chinese	Gene expression data of HBV-related HCC and paired non-tumor tissues	|log2(foldchange)| ≥ 1, *P* < 0.01
TCGA-LIHC	Illumina HiSeq	374/50	mixed Asian/European origin	Gene expression RNAseq of tumor and normal tissues	|log2(foldchange)| ≥ 1, *P* < 0.01
GSE76903	Illumina HiSeq	20/20	Chinese	miRNA-seq of matched adjacent normal and primary tumor samples from HCC patients	average normalized count ≥ 1

*TCGA-LIHC* The Cancer Genome Atlas Liver Hepatocellular Carcinoma

**Table 4:** The three up-regulated exonic circRNAs and the expression of their parent genes in TCGA-LIHC and GSE14520-GPL3921

ID in circBase	Position	Strand	Genomic length	Spliced length	CircRNA regulation	Parent gene symbol	Parent gene regulation in TCGA-LIHC	Parent gene regulation in GSE14520	CircRNA vs. parent gene regulation direction
hsa_circ_0009910	chr1:12,049,221–12,052,747	+	3526	315	Up	MFN2	Up	No significant	Different
hsa_circ_0049783	chr19:14,705,328–14,705,612	+	284	126	Up	CLEC17A	No significant	Not available	Different
hsa_circ_0089172	chr9:134,049,441–134,053,797	+	4356	526	Up	NUP214	Up	No significant	Different

We used divergent primers specifically amplifying circRNA in cDNA but not genomic DNA, and convergent primers amplifying both circRNA and linear RNA and in cDNA and parental gene in genomic DNA to confirm that hsa_circ_0009910 and hsa_circ_0089172 were indeed circular (Fig. 2g). Following RNase R treatment, expression levels of hsa_circ_0009910 and hsa_circ_0089172 were almost unaffected. In contrast, linear β-actin was substantially digested, demonstrating that hsa_circ_0009910 and hsa_circ_0089172 were resistant to RNase R treatment and were stable transcripts (Fig. 2h). Agarose gel electrophoresis following circRNAs amplification showed that these two circRNAs existed in the culture medium of tumor cells but not in the control group, indicating hsa_circ_0009910 and hsa_circ_0089172 excretion from hepatoma cells (Fig. 2i).

### Identification of four circRNA–miRNA interactions

**Fig. 3:**
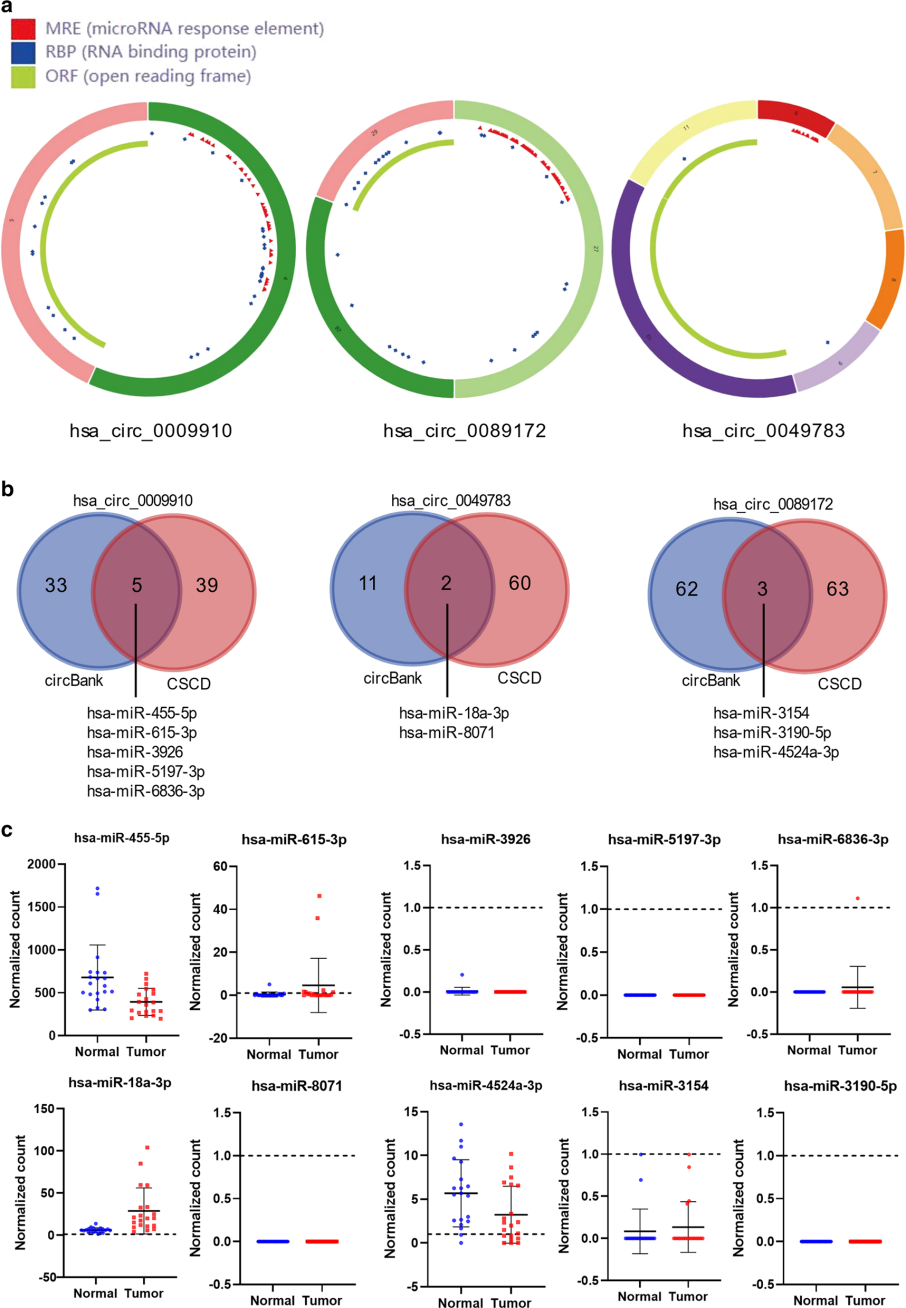
Identification of circRNA–miRNA interactions.** a** Structural patterns of the three circRNAs by the Cancer-specific circRNAs database (CSCD).** b** Prediction of miRNA binding by circBank and CSCD.** c** Expression of the ten candidate miRNAs in the miRNA-seq dataset GSE76903

To depict whether the three up-regulated circRNAs could regulate gene expression by functioning as miRNA sponges in HCC, we collected their basic structural patterns from the CSCD database, exhibiting numerous miRNA response elements (MREs; Fig. 3a). We further collected their potential miRNA bindings by intersecting the MREs predicted by two web tools, CSCD and circbank (Fig. 3b). A total of five miRNAs (hsa-miR-455-5p, hsa-miR-615-3p, hsa-miR-3926, hsa-miR-5197-3p, and hsa-miR-6836-3p) were identified for hsa_circ_0009910, two miRNAs (hsa-miR-18a-3p and hsa-miR-8071) for hsa_circ_0049783, and three miRNAs (hsa-miR-4524a-3p, hsa-miR-3154, hsa-miR-3190-5p) for hsa_circ_0089172. Among these miRNAs, 4 (hsa-miR-455-5p, hsa-miR-615-3p, hsa-miR-18a-3p, hsa-miR-4524a-3p) are expressed in primary HCC or matched adjacent normal tissues (average normalized count ≥ 1) according to a recent study providing genome-wide miRNA expression profile (Fig. 3c; Table 5). Thus, a total of four circRNA–miRNA interactions, including three circRNAs and four miRNAs were identified.

**Table 5:** The target miRNAs of three circRNAs predicted by circBank and CSCD

circRNA	miRNA	miRanda binding position	MSA Start	MSA End	Site Type	Expressed in primary HCC or matched adjacent normal tissues according to GSE76903*
hsa_circ_0009910	hsa-miR-455-5p	273	23	29	7mer-m8	Yes
	hsa-miR-615-3p	266	15	22	8mer-1a	Yes
	hsa-miR-3926	296	43	49	7mer-m8	No
	hsa-miR-5197-3p	1	65	71	7mer-m8	No
	hsa-miR-6836-3p	270	20	26	7mer-m8	No
hsa_circ_0049783	hsa-miR-18a-3p	12	78	84	7mer-m8	Yes
	hsa-miR-8071	97	62	68	7mer-1a	No
hsa_circ_0089172	hsa-miR-4524a-3p	160	57	63	7mer-m8	Yes
	hsa-miR-3154	494	33	40	8mer-1a	No
	hsa-miR-3190-5p	397	50	55	6mer	No

* average normalized count ≥ 1

### Construction of circRNA–miRNA–mRNA network

**Fig. 4:**
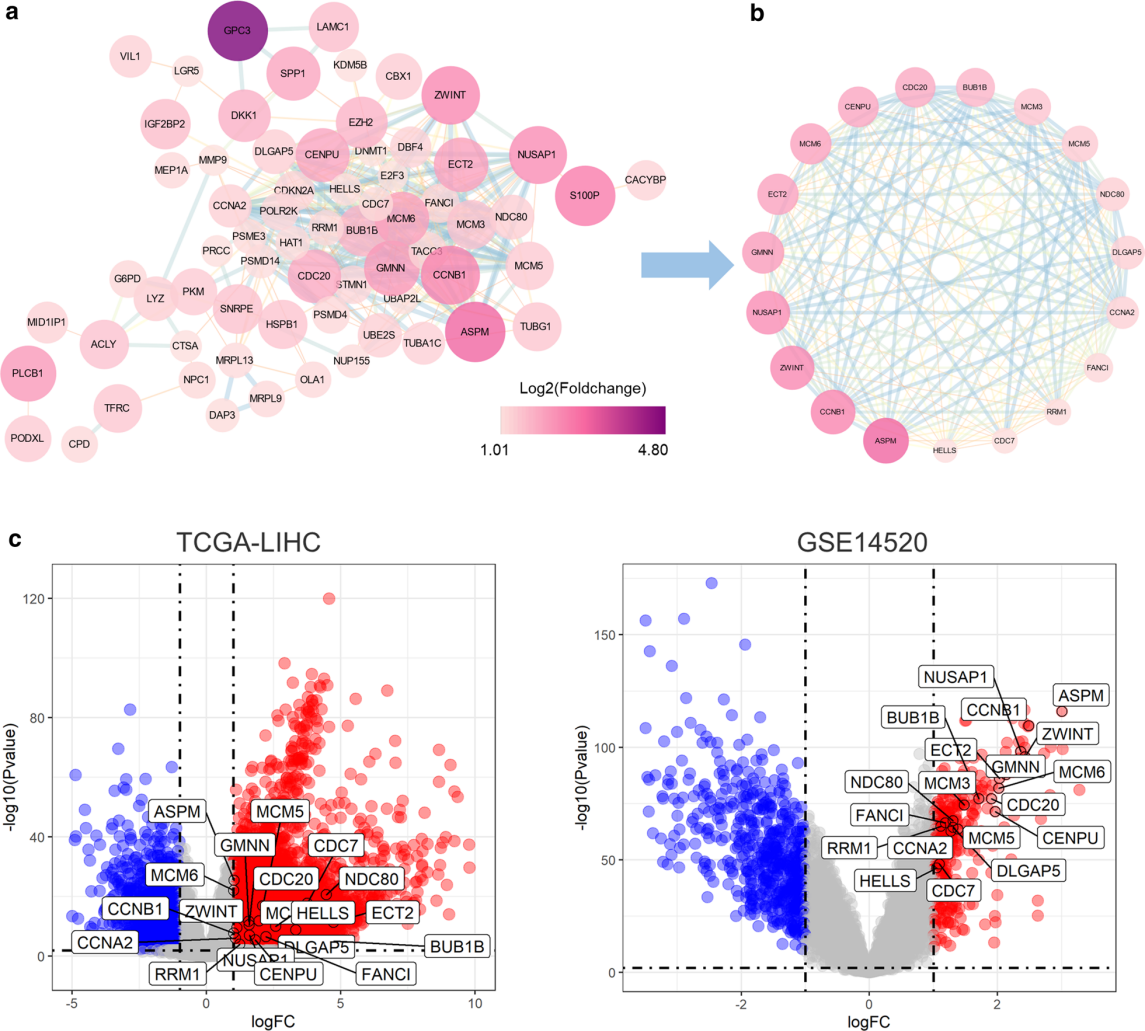
Construction of circRNA–miRNA–mRNA regulatory network.** a** Volcano plot of significantly differentially expressed genes in TCGA-LIHC (left) and GSE14520 (right).** b** Venn diagram shows the intersections between miRNA targets predicted by miRWalk, significantly up-regulated genes from TCGA-LIHC and GSE14520.** c** A circRNA–miRNA–mRNA network consisting of three cricRNAs, four miRNAs, and 95 genes

A total of 12,908 target genes of the four miRNAs mentioned above were obtained from miRWalk, a web tool for predicting miRNA binding sites. Additionally, 6648 genes were significantly up-regulated in tumors compared with normal tissues from The Cancer Genome Atlas Liver Hepatocellular Carcinoma (TCGA-LIHC) with mixed Asian/European origin, while 337 increased genes from GSE14520 dataset from a Chinese cohort (Fig. 4a). To further narrow the scope, we intersected the miRNA targets predicted by miRWalk with the significantly up-regulated genes determined from TCGA-LIHC and GSE14520, resulting in a total of 95 candidate genes for further analysis (Fig. 4b). By integrating the circRNA-miRNA and miRNA-mRNA interactions, we established and visualized a circRNA–miRNA–mRNA regulatory network in HCC (Fig. 4c).

### Identification of 19 hub genes

**Fig. 5:**
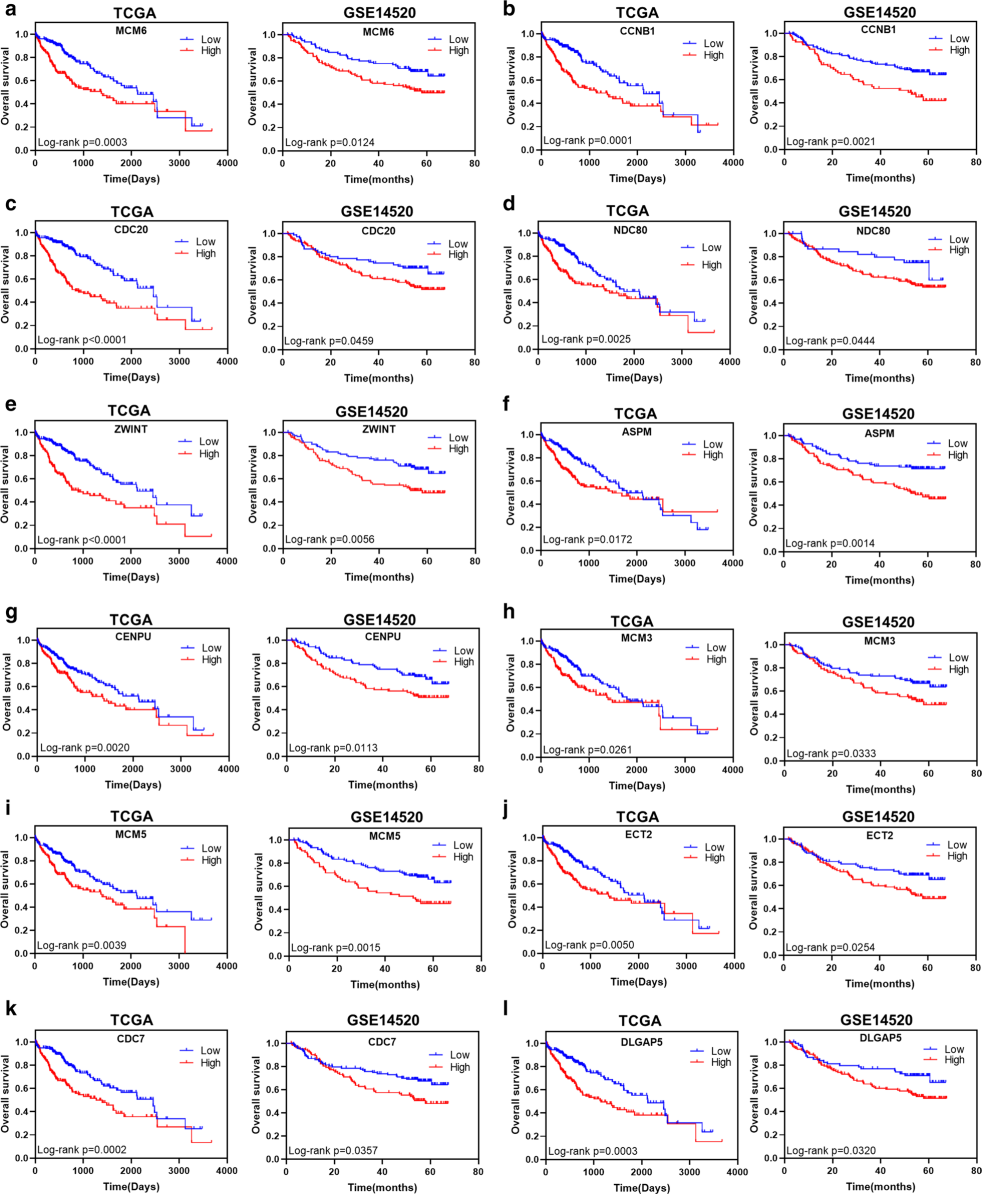
Identification of 19 hub genes from the interaction network.** a** A PPI network of the 95 candidate genes consists of 67 nodes and 358 edges. The node size varies from small to large according to the number of neighbored genes.** b** A subnetwork consists of 19 nodes and 166 edges extracted from (**a**) with the MCODE clustering algorithm.* PPI* protein-protein interaction.** c** Volcano plot showing the 19 hub genes in TCGA-LIHC (left) and GSE14520 (right) datasets

Figuring out the biological function of potential circRNA-related genes could predict the corresponding function of circRNAs. Interactions among the 95 candidate genes were predicted with the STRING tool, leading to a network containing 67 nodes and 358 edges after removing unconnected nodes (Fig. 5a). Subsequently, we employed the MCODE clustering algorithm to find hub genes from the interaction network. One subnetwork with 19 significantly up-regulated nodes and 166 edges was identified with the k-core = 2 (Fig. 5b, c, and Table 2), suggesting the 19 hub genes’ critical roles in HCC.

### Twelve hub genes with prognostic values

**Fig. 6:**
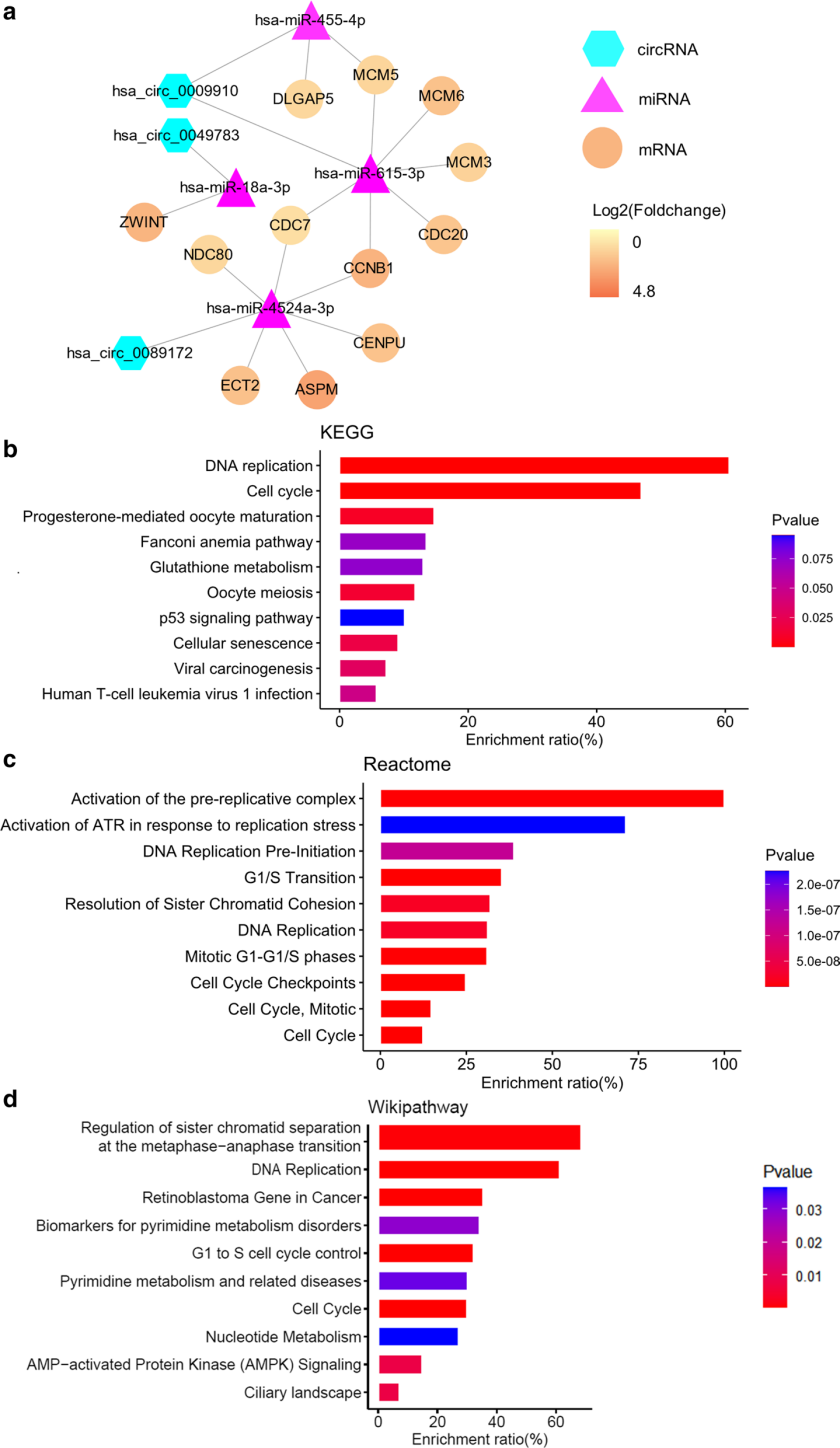
Kaplan-Meier’s survival curves showed the correlations between expression levels of 12 hub genes and reduced overall survival. A log-rank test was used

To investigate the prognostic values of the hub genes, survival analysis was performed on patients from TCGA-LIHC and GSE14520. Kaplan-Meier’s survival curves showed that among the 19 hub genes, high expression of 12 genes, including MCM6, CCNB1, CDC20, NDC80, ZWINT, ASPM, CENPU, MCM3, MCM5, ECT2, CDC7, and DLGAP5 was significantly associated with poorer overall survival (Log-rank *P* < 0.05) in patients from TCGA-LIHC and GSE14520 (Fig. 6; Table 2). Correlations between expression levels of these 12 hub genes and reduced disease-free interval, disease-specific survival, and progression-free interval were also observed in patients from TCGA-LIHC (hazard ratio > 1; Table 6). A circRNA–miRNA–hub gene network consisting of four regulatory modules (hsa_circ_0009910–hsa-miR-455-5p–DLGAP5/MCM5 axis, hsa_circ_0009910–hsa-miR-615-3p–MCM5/MCM6/MCM3/CDC20/CCNB1/CDC7 axis, hsa_circ_0049783–hsa-miR-18a-3p–ZWINT axis, and hsa_circ_0089172–hsa-miR-4524a-3p–CDC7/CCNB1/CENPU/ASPM/ECT2/NDC80 axis) was then built to delineate the links among the identified circRNAs, miRNAs and hub genes (Fig. 6a).

**Table 6:** Survival analysis for 12 significantly up-regulated hub genes in TCGA-LIHC

Hubgenes	DFI		DSS		PFI	
	HR(95 %CI)	Log-rank *P* value	HR(95 %CI)	Log-rank *P* value	HR(95 %CI)	Log-rank *P* value
MCM6	1.923(1.358–2.724)	< 0.0001****	1.901(1.217–2.971)	0.0040**	1.865(1.386–2.510)	< 0.0001****
CCNB1	1.847(1.323–2.578)	0.0002***	2.189(1.404–3.412)	0.0005***	1.947(1.447–2.619)	< 0.0001****
CDC20	1.614(1.144–2.278)	0.0038**	3.028(1.936–4.736)	< 0.0001****	1.809(1.345–2.434)	< 0.0001****
NDC80	1.497(1.077–2.081)	0.0155*	1.786(1.145–2.786)	0.0098**	1.501(1.118–2.015)	0.0061**
ZWINT	2.076(1.466–2.938)	< 0.0001****	2.170(1.390–3.389)	0.0006***	1.899(1.377–2.499)	< 0.0001****
ASPM	1.419(1.022–1.972)	0.0360*	1.794(1.135–2.767)	0.0104*	1.432(1.068–1.920)	0.0158*
CENPU	1.497(1.077–2.081)	0.0155*	1.786(1.145–2.786)	0.0098**	1.501(1.118–2.015)	0.0061**
MCM3	1.615(1.160–2.248)	0.0039**	1.734(1.109–2.711)	0.0131*	1.627(1.210–2.186)	0.0010**
MCM5	1.372(0.986–1.908)	0.0576	1.623(1.040–2.532)	0.0305*	1.446(1.076–1.942)	0.0128*
ECT2	1.488(1.070–2.070)	0.0168*	2.084(1.338–3.245)	0.0013**	1.564(1.166–2.098)	0.0026**
CDC7	1.255(0.902–1.745)	0.1726	2.151(1.376–3.364)	0.0006***	1.453(1.073–1.967)	0.0128*
DLGAP5	1.484(1.067–2.064)	0.0177*	2.262(1.452–3.525)	0.0004***	1.671(1.245–2.243)	0.0005***

*HR* hazard ratio,* DFI* disease-free interval,* DSS* disease specific survival,* PFI* progression free interval

### Functional enrichment analysis of hub genes

**Fig. 7:**
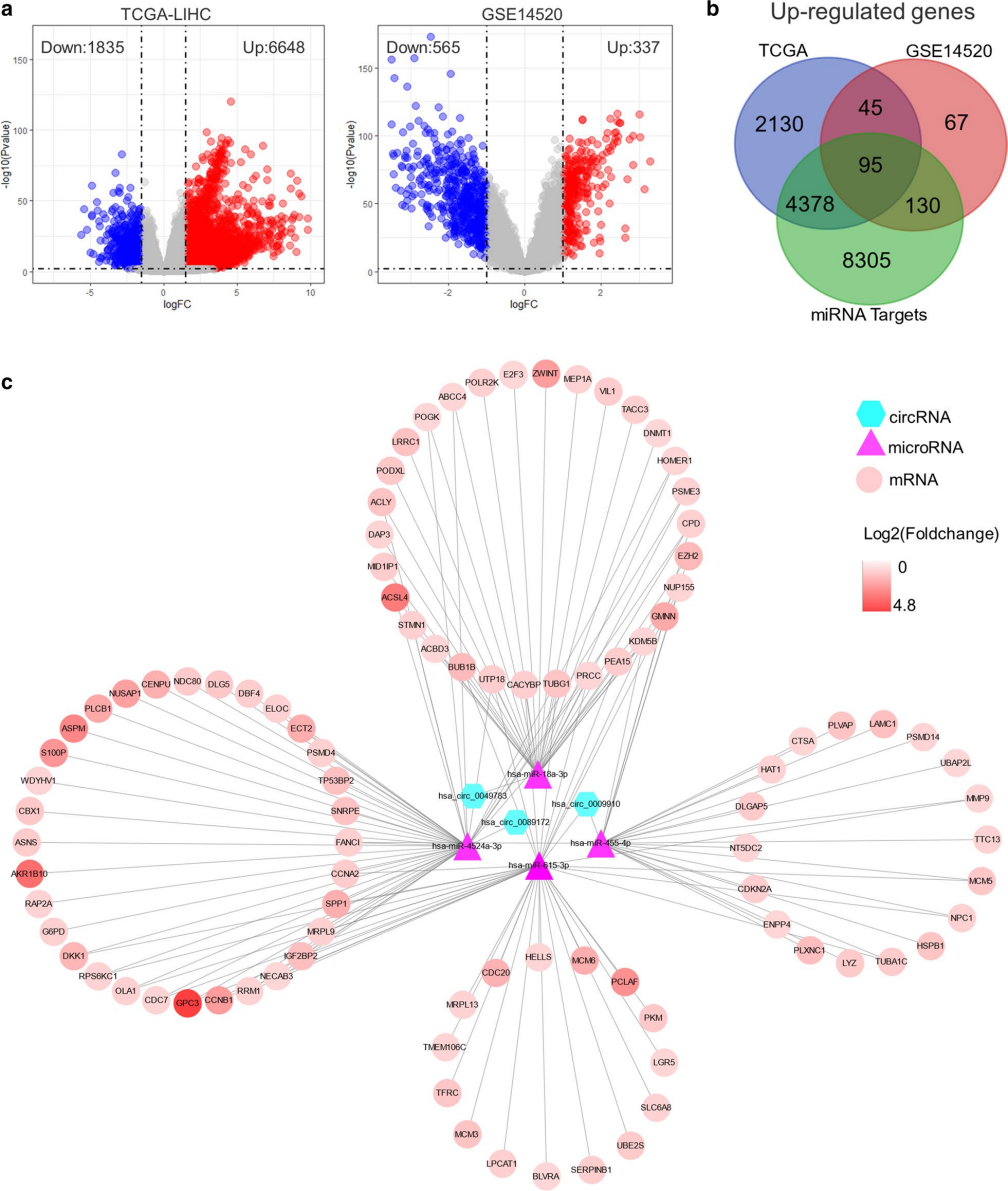
CircRNA–miRNA–hub gene network and pathway enrichment analysis. **a** A CircRNA–miRNA–hub gene network consisting of three cricRNAs, four miRNAs, and 12 hub genes.** b-d** Enrichment analysis of the 12 survival-related hub genes.** b** KEGGpathway.** c** Reactome pathway.** d** Wikipathway

Enrichment analysis was performed to illustrate the functional annotations of the 12 survival-related hub genes. KEGG pathway enrichment analysis indicated that hub genes were mainly enriched in DNA replication and cell cycle. For the Reactome pathways, the hub genes were enriched in activation of the pre-replicative complex, G1/S transition, resolution of sister chromatid cohesion, and DNA replication. For the Wikipathway, enrichment of the hub genes in regulation of sister chromatid separation at the metaphase-anaphase transition, DNA replication, and retinoblastoma gene in cancer was identified (Fig. 7b-d).

## Discussion

In recent years, the application of large-scale gene expression profiling and the development of bioinformatics has allowed us to systematically uncover the molecular regulation mechanisms associated with tumorigenesis and development. However, large sample sizes are required to identify circRNA expression differences with greater statistical power [30]. In this study, we obtained three up-regulated circRNAs, namely hsa_circ_0009910, hsa_circ_0049783, and hsa_circ_0089172, through jointly analyzing of three tissue microarray datasets and a blood microarray dataset, suggesting these deregulated circRNAs in HCC tissues could be secreted into plasma [28]. Recent studies found that hsa_circ_0009910 was overexpressed in osteosarcoma and ovarian cancer cells, acting as a sponge of miR-449a and promoting the expression of miR-449a target IL6R in osteosarcoma while suppressing miR-145 in ovarian cancer cells [31, 32]. The expression of hsa_circ_0089172 was up-regulated in Hashimoto’s thyroiditis patients and might function via sponging miR-125a-3p [33]. However, the role of hsa_circ_0049783 has not been reported.

To explore the interaction of the candidate circRNAs with miRNA and mRNA, we constructed a circRNA–miRNA–mRNA regulatory network based on the mechanism that circRNAs harboring miRNA binding sites might act as miRNA sponges to regulate downstream gene expression [34]. A total of four miRNAs, namely hsa-miR-455-5p, hsa-miR-615-3p, hsa-miR-18a-3p, and hsa-miR-4524a-3p, were screened out to interact with the candidate circRNAs. Cumulative evidence indicates that hsa-miR-455-5p functions as a tumor suppressor in cancer progression of stomach and prostate [35, 36]. Hsa-miR-615-3p has also been reported as a tumor suppressor in cancers of lung and esophagus [37]. Downstream hub genes, including MCM6, CCNB1, CDC20, NDC80, ZWINT, ASPM, CENPU, MCM3, MCM5, ECT2, CDC7, and DLGAP5, were up-regulated and associated with reduced survival in two HCC cohorts. Our enrichment analysis showed that these hub genes were mainly enriched in DNA replication and cell cycle, indicating their critical roles in the pathogenesis of HCC. Consistently, Jia et al. suggested that MCM6 could be an optimal diagnostic biomarker and a potential therapeutic target for HCC in a Southern Chinese Zhuang population [38]. Ju et al. proved that the expression level of NDC80 was significantly elevated in HCC tissues and contributed to tumor progression [39]. Ying et al. reported that ZWINT was overexpressed in HCC samples and associated with poor overall survival and tumor recurrence [40]. Lin et al. suggested that ASPM overexpression was a marker for enhanced vascular invasive, metastatic potential, and poor prognosis of HCC [41]. Chen et al. identified that the ECT2 upregulation was associated with early HCC recurrence and poor survival [42]. Liao et al. reported that DLGAP5 was significantly up-regulated in HCC specimens and promoted cell proliferation [43].

##  Conclusions

In summary, we screened out three novel circRNAs up-regulated both in tissue and plasma from HCC patients and constructed a regulatory mechanism of circRNA–miRNA–mRNA interaction by integrating multiple expression profiles and bioinformatics tools. Our study provides a new perspective on the effectiveness of the candidate circRNAs as biomarkers and clinical treatment targets for HCC.

## Data Availability

All data generated or analysed during this study are included in this published article.
