# Correction to: Three novel circRNAs upregulated in tissue and plasma from hepatocellular carcinoma patients and their regulatory network

**DOI:** 10.1186/s12935-021-01788-0

**Published:** 2021-02-03

**Authors:** Lianghai Wang, Lisha Zhou, Jun Hou, Jin Meng, Ke Lin, Xiangwei Wu, Xueling Chen

**Affiliations:** Key Laboratory of Xinjiang Endemic and Ethnic Diseases/the First, Affiliated Hospital, Shihezi University School of Medicine, Shihezi, Xinjiang China

## Correction to: Cancer Cell Int (2021) 21:72 https://doi.org/10.1186/s12935-021–01762-w

Following publication of the original article [1], we were notified that Figs. 4 to 7 were misplaced: Fig. 4 in the online version should have been Fig. 5, Fig. 5 in the online version should have been Fig. 6, Fig. 6 in the online version should have been Fig. 7, and Fig. 7 in the online version should have been Fig. 4. Figure citations and figure legends were published in the correct order.

The original article has been corrected.
